# Tristetraprolin regulation of interleukin-22 production

**DOI:** 10.1038/srep15112

**Published:** 2015-10-21

**Authors:** Lorena Härdle, Malte Bachmann, Franziska Bollmann, Andrea Pautz, Tobias Schmid, Wolfgang Eberhardt, Hartmut Kleinert, Josef Pfeilschifter, Heiko Mühl

**Affiliations:** 1 pharmazentrum frankfurt/ZAFES, University Hospital Goethe-University Frankfurt, Germany; 2 Department of Pharmacology, University Medical Center of the Johannes-Gutenberg University Mainz, Mainz, Germany; 3 Institute of Biochemistry I-Pathobiochemistry, Faculty of Medicine, Goethe-University Frankfurt, Germany

## Abstract

Interleukin (IL)-22 is a STAT3-activating cytokine displaying characteristic AU-rich elements (ARE) in the 3′-untranslated region (3′-UTR) of its mRNA. This architecture suggests gene regulation by modulation of mRNA stability. Since related cytokines undergo post-transcriptional regulation by ARE-binding tristetraprolin (TTP), the role of this destabilizing protein in IL-22 production was investigated. Herein, we demonstrate that TTP-deficient mice display augmented serum IL-22. Likewise, IL-22 mRNA was enhanced in TTP-deficient splenocytes and isolated primary T cells. A pivotal role for TTP is underscored by an extended IL-22 mRNA half-life detectable in TTP-deficient T cells. Luciferase-reporter assays performed in human Jurkat T cells proved the destabilizing potential of the human IL-22-3′-UTR. Furthermore, overexpression of TTP in HEK293 cells substantially decreased luciferase activity directed by the IL-22-3′-UTR. Transcript destabilization by TTP was nullified upon cellular activation by TPA/A23187, an effect dependent on MEK1/2 activity. Accordingly, IL-22 mRNA half-life as determined in TPA/A23187-stimulated Jurkat T cells decreased under the influence of the MEK1/2 inhibitor U0126. Altogether, data indicate that TTP directly controls IL-22 production, a process counteracted by MEK1/2. The TTP-dependent regulatory pathway described herein likely contributes to the role of IL-22 in inflammation and cancer and may evolve as novel target for pharmacological IL-22 modulation.

Interleukin (IL)-22^1^,^2^ is a member of the IL-10 cytokine family sharing some fundamental structural and biological properties with IL-10, IL-20, IL-24, and IL-6. Biochemically, this is exemplified by the shared ability of aforementioned cytokines to mediate robust activation of the transcription factor signal transducer and activator of transcription (STAT)-3 and associated STAT3-dependent downstream events connecting to proliferation, anti-apoptosis, strengthening of host-defense, and regulation of inflammatory responses. A particularly striking feature of IL-22 is that this cytokine specifically targets epithelial (-like) cells, among others keratinocytes and hepatocytes as well as lung and intestinal epithelial cells. Restricted expression of the decisive IL-22 receptor chain IL-22R1 on aforementioned cell types is regarded the biological basis for this selectivity^3^,^4^,^5^,^6^. Leukocytic cells generally neither express IL-22R1 nor respond to IL-22. Yet, IL-22 is largely a lymphocyte-derived cytokine being efficiently produced by natural killer and related innate lymphoid cells, by invariant NK-T and γδT cells and a broad array of adaptive CD4^+^ or CD8^+^ T cells, the former including differentiated Th1, Th17, and Th22 subsets^4^,^7^,^8^,^9^,^10^,^11^.

The role of IL-22 in disease is truly context dependent. IL-22 exerts tissue-protective/anti-microbial functions in infection- and/or injury-driven diseases at biological barriers such as intestine, lung, and liver^5^. Examples of pathological conditions with IL-22 displaying protective properties include intestinal infection by *Citrobacter rodentium*^12^, colitis induced by dextran sulfate sodium^13^, concanavalin A^14^- or acetaminophen-induced acute liver injury^15^ as well as ventilator-induced lung injury^16^ and mucosal candidiasis^17^. However, Janus-faced IL-22 also shows the potential to aggravate some aspects of pathological inflammation. Specifically, IL-22 promotes disease in experimental psoriasis^18^ and arthritis^19^. Besides induction of inflammatory chemokines, a major mode of IL-22 disease-promoting functions in this context is its pro-proliferative and anti-apoptotic action targeting keratinocytes and synoviocytes, respectively. It is obvious that those two key properties likewise relate to the unfortunate role of IL-22 in cancer^20^.

Given the multilayered biological functions of IL-22, knowledge of molecular mechanisms driving its production is crucial. Previous reports indicate that transcription factors/nuclear receptors such as STAT3, retinoid orphan receptor-γt and aryl hydrocarbon receptor^21^ as well as the cAMP response element-binding protein and nuclear factor of activated T cells (NF-AT)^22^ are involved in initiation of IL-22 gene transcription. However, firm knowledge on post-transcriptional molecular mechanisms regulating IL-22 expression is lacking. Sequence analysis reveals a remarkable density of adenylate- and uridylate (AU)-rich elements (ARE) in the 3′-untranslated region (3′-UTR) of human and murine IL-22 mRNA (Fig. 1). The presence of those elements at this location suggests post-transcriptional regulation by modulation of mRNA stability^23^,^24^,^25^. The CCCH zinc finger protein tristetraprolin (TTP)^25^ has been identified as crucial trans-acting factor binding to ARE within the 3′-untranslated region of labile mRNA molecules. Subsequent to target binding, TTP is supposed to destabilize mRNA molecules by enforcing the processes of mRNA deadenylation and decapping thereby augmenting decay by exonucleases^24^,^25^. Cytokines are a prime target of TTP biological activity. A well characterized example is tumor necrosis factor (TNF)-α, the expression of which is most efficiently controlled by TTP as part of a negative feedback loop aiming at control of exacerbated inflammation and/or to initiate its resolution^26^,^27^. Since mRNA expression of IL-22-related IL-10^25^,^28^ and IL-6^25^,^29^ is known to be modulated by TTP and post-transcriptional gene regulation is frequently organized in functional units^30^, we set out to investigate in detail the role of TTP in IL-22 expression.

## Results

### Enhanced production of IL-22 detected in TTP-deficient mice and *ex vivo* stimulated TTP^−/−^ splenocytes

TTP^−/−^ mice display a characteristic inflammatory syndrome with erosive arthritis, conjunctivitis, dermatitis, and cachexia as obvious severe symptoms^31^. In accord with the picture of uncontrolled persistent inflammation, we report for the first time on significantly elevated systemic levels of IL-22 in TTP^−/−^ mice as compared to wildtype littermates (Fig. 2a). Likewise, serum levels of the IL-22-related and TTP-regulated^25^,^28^,^29^ cytokines IL-6 (Fig. 2b) and IL-10 (Fig. 2c) were increased. Data are in accord with previously reported IL-22 mRNA upregulation as detected in skin and draining lymph nodes of TTP^−/−^ mice^32^.

In order to further investigate on a cellular level IL-22 production in the context of TTP deficiency, cytokine production by *ex vivo* stimulated splenocytes was assessed. For that purpose, cytokine- (exposure to IL-12/IL-18) and T cell receptor (TCR)- (exposure to αCD3/αCD28) stimulated IL-22 release was evaluated in splenocytes isolated from TTP^−/−^ mice and respective wildtype littermates. Of note, IL-18, particularly in combination with IL-12, is a most potent mediator of cytokine-based T cell activation^33^. Here we demonstrate that IL-12/IL-18- (Fig. 3a, left panel) and αCD3/αCD28- (Fig. 3b) mediated IL-22 production was potentiated in splenocytes derived from TTP^−/−^ mice. Likewise, production of IL-6 and IL-10, determined in splenocytes exposed to IL-12/IL-18, was markedly increased in TTP^−/−^ mice (Fig. 3a, middle and right panel). Altogether, data relate TTP deficiency to enhanced IL-22 production as detected *in vivo* and on cell culture level.

### TTP deficiency associates with augmented IL-22 mRNA half-life as detected in primary murine CD3^+^ T cells

In order to more directly relate TTP expression with IL-22 mRNA stability in T cells, actinomycin D experiments were performed using isolated splenic CD3^+^ T cells from TTP^−/−^ mice or wildtype littermates, respectively. IL-22 mRNA induction was achieved by activating T cells with αCD3/αCD28. Notably, a 4 h incubation period was sufficient to mediate robust induction of IL-22 mRNA under those experimental conditions. In accord with aforementioned observations, T cells obtained from TTP^−/−^ mice displayed significantly enhanced IL-22 mRNA expression as compared to wildtype littermates (Fig. 4a). Actinomycin D experiments performed subsequent to this 4 h gene induction period revealed an IL-22 mRNA half-life of approximately 29 minutes that was increased upon TTP deficiency (Fig. 4b). Data altogether demonstrate a role for TTP in the regulation of IL-22 mRNA stability.

### The destabilizing potential of the IL-22-3′-UTR

**Figure 1:**
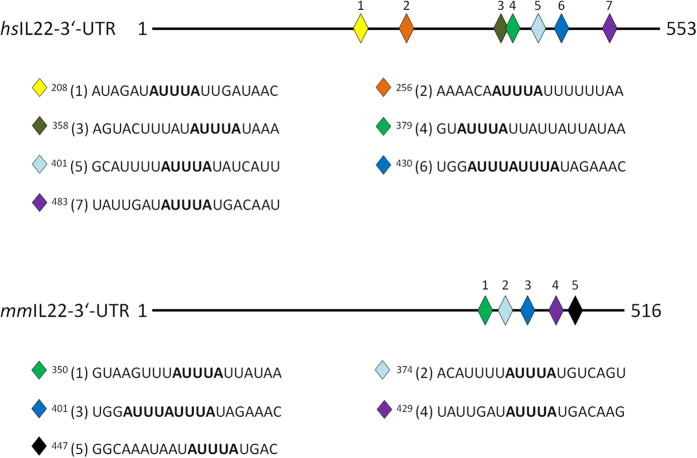
The IL-22-3′-UTR harbors several AU-rich elements (ARE). Schematic of the human and murine IL-22-3′-UTR. Positions of ARE are indicated by numbered rhombi and corresponding sequences are shown below. Equal rhombus colors depict similar motifs in the human and mouse IL-22-3′-UTR. Superscript numbers indicate the starting nucleotide position of a specific ARE within the IL-22-3′-UTR.

**Figure 2:**
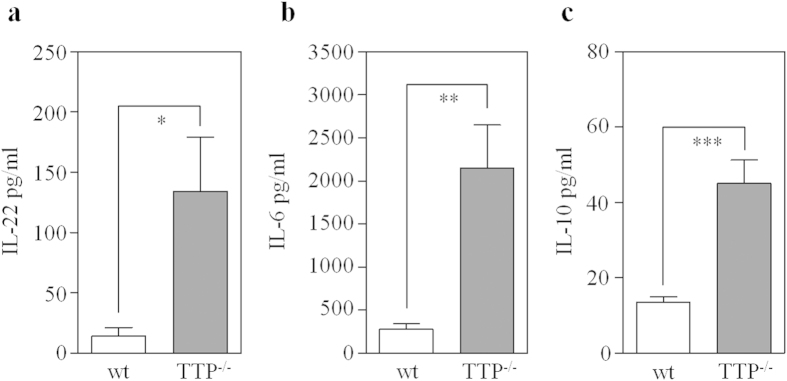
Elevated serum IL-22 levels detected in TTP^−/−^ mice. (**a**) IL-22 (wildtype (wt), n = 6; TTP^−/−^, n = 5; *p = 0.0178), (**b**) IL-6 (wt, n = 6; TTP^−/−^, n = 7; **p = 0.0054) and (**c**) IL-10 (wt, n = 9; TTP^−/−^, n = 6; ***p < 0.001) protein levels were determined in the serum of 14–16 week-old TTP^−/−^ mice and their wt littermates by ELISA. Data are expressed as means ± SEM. Statistical analysis, Student’s t-test.

**Figure 3:**
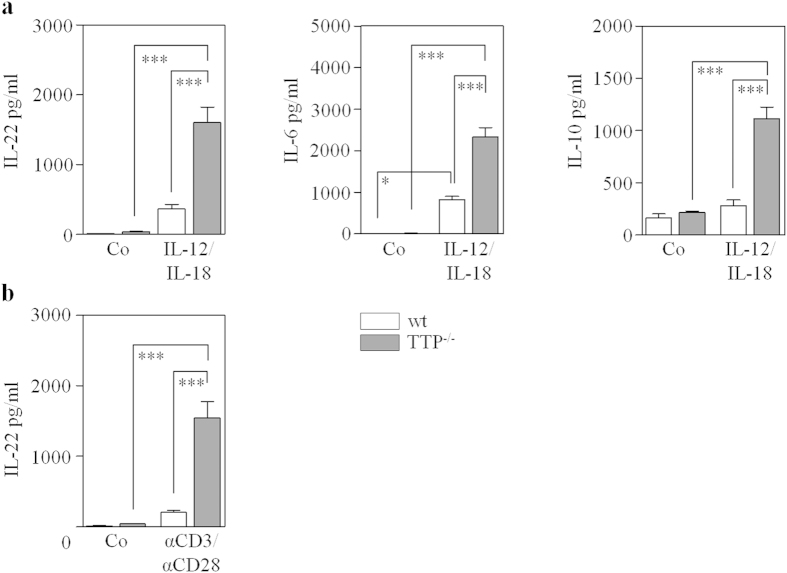
Increased IL-22 production by *ex vivo* stimulated splenocytes derived from TTP^−/−^ mice. (**a**,**b**) Splenocytes were isolated from TTP^−/−^ mice (n = 4, grey bars) and wildtype littermates (n = 3, open bars). (**a**) Cells of individual mice were either kept as unstimulated control (Co) or stimulated with IL-12 (10 ng/ml)/IL-18 (50 ng/ml). After 24 h, IL-22 (left panel), IL-6 (middle panel), and IL-10 (right panel) secretion was determined by ELISA. (**b**) Splenocytes were stimulated with αCD3 (15 μg/ml)/αCD28 (1.5 μg/ml). After 24 h, IL-22 secretion was determined by ELISA. (**a**,**b**) Data are shown as means ± SEM (*p < 0.05, ***p < 0.001). Statistical analysis, one-way analysis of variance with *post-hoc* Bonferroni correction.

**Figure 4:**
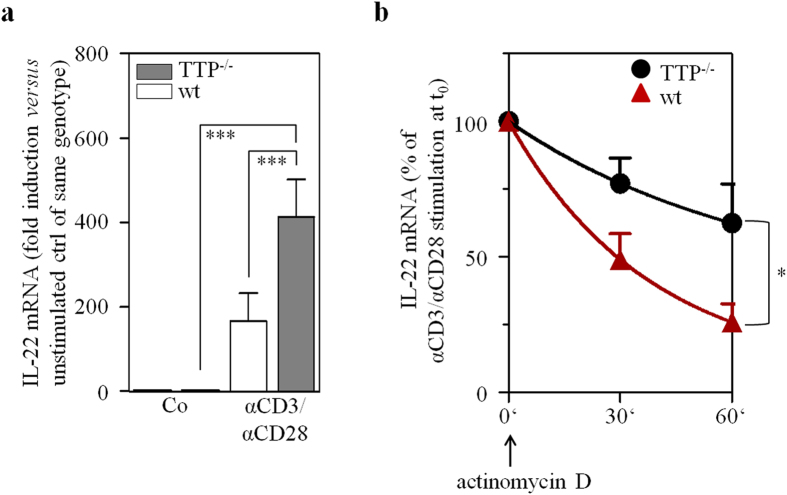
Splenic CD3^+^ T cells from TTP^−/−^-mice display prolonged IL-22 mRNA half-life. (**a**) Splenic CD3^+^ T-cells were isolated from TTP^−/−^ mice (n = 14) and wildtype (wt) littermates (n = 11). Cells of individual mice were either kept as unstimulated control (Co) or stimulated with αCD3 (15 μg/ml)/αCD28 (1.5 μg/ml). After 4 h, IL-22 mRNA was determined by realtime PCR. IL-22 mRNA was normalized to that of GAPDH (means ± SEM; ***p < 0.001; statistical analysis performed on raw data, one-way analysis of variance with *post-hoc* Bonferroni correction). (**b**) Splenic CD3^+^ T-cells of individual TTP^−/−^ mice (n = 10) and wt littermates (n = 15) were stimulated with αCD3 (15 μg/ml)/αCD28 (1.5 μg/ml). After 4 h, actinomycin D (10 μg/ml) was added and IL-22 mRNA levels were determined by realtime PCR at the indicated time points. All cultures were adjusted to a final concentration of 0.05% DMSO (vehicle for actinomycin D). IL-22 mRNA was normalized to that of GAPDH (means ± SEM depicted as [% of IL-22 mRNA expression at t_0_, the time point of actinomycin D addition]; *p = 0.029). Statistical analysis on percent data, Student’s t-test.

**Figure 5:**
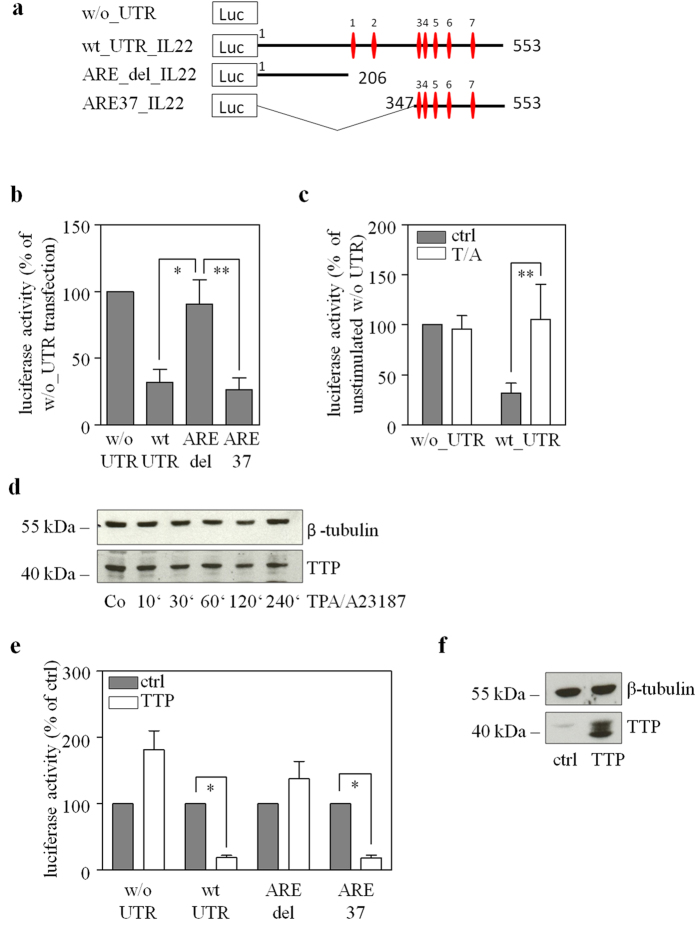
The mRNA destabilizing potential of the IL-22-3′-UTR as detected in luciferase reporter assays. (**a**) Schematic of luciferase reporter constructs. The human IL-22-3′-UTR or indicated fragments were cloned downstream of a luciferase gene driven by the constitutively active SV40-promoter. Rhombi indicate ARE. w/o_UTR, construct without IL-22-3′-UTR sequence; wt_UTR_IL22, construct with the complete IL-22-3′-UTR; ARE_del_IL22, construct with the proximal IL-22-3′-UTR sequence lacking ARE; ARE37_IL22, construct with the distal IL-22-3′-UTR sequence including ARE. (**b**) Jurkat T cells were transfected with indicated luciferase reporter plasmids. After 16 h, luciferase activity was determined. Data depicted (as % of w/o_UTR-transfected cells) are expressed as means ± SD (n = 3); *p < 0.05, ** p < 0.01. Statistical analysis on percent data, one-way analysis of variance with *post-hoc* Bonferroni correction. (**c**) Jurkat T cells were transfected with indicated luciferase reporter plasmids. After 16 h of rest, cells were left untreated (ctrl) or stimulated with TPA (T, 100 ng/ml)/A23187 (A, 10 μM) for 4 h. All cultures were adjusted to a final concentration of 0.11% DMSO (vehicle for T/A). Luciferase activity is depicted (as % of w/o_UTR-transfected ctrl cells) and expressed as means ± SD (n = 3); **p < 0.01. Statistical analysis on percent data, one-way analysis of variance with *post-hoc* Bonferroni correction. (**d**) Jurkat T cells were kept as unstimulated control or stimulated with TPA (T, 100 ng/ml)/A23187 (A, 10 μM). All cultures were adjusted to a final concentration of 0.11% DMSO (vehicle for T/A). At indicated time points, TTP protein expression was determined by immunoblot analysis. One representative of three independently performed experiments is shown. (**e**) HEK293 cells were transfected for 16 h with indicated luciferase reporter plasmids together with either a TTP-expression (pZEO_hTTP)- or a control-plasmid (ctrl). Luciferase activity is depicted (as % of cells transfected with the same luciferase reporter plasmid plus ctrl-plasmid) and expressed as means ± SD (n = 4; *p = 0.02 for wt_UTR and ctrl or TTP, *p = 0.028 for ARE37 and ctrl or TTP). Statistical analysis on raw data, Student’s t-test. (**f**) TTP overexpression was confirmed by immunoblot analysis of lysates obtained from (**e**). One representative of four independently performed experiments is shown.

Luciferase-reporter assays were performed in Jurkat T and HEK293 cells to investigate mechanisms regulating IL-22 mRNA stability in detail. Since murine and human IL-22-3′-UTR ARE sequences display extensive homology (Fig. 1) and aforementioned cells lines are of human origin, further experiments were performed using human IL-22-3′-UTR sequences. For that purpose, luciferase-reporter constructs were transfected with variants of the human IL-22-3′-UTR cloned next to a luciferase-reporter gene (Fig. 5a). Since luciferase enzyme activity can be readily determined, this experimental approach allows to straightforwardly evaluate the potential for post-transcriptional regulation by ARE originally located in the IL-22-3′-UTR. Using human Jurkat T cells, we demonstrate that the full length IL-22-3′-UTR (transfection of wt_UTR_IL22) in fact displays mRNA destabilizing characteristics. Transfection of ARE37_IL22, containing a 206 nt ARE-rich deletion fragment representing the distal part of the IL-22-3′-UTR, likewise reduced luciferase-reporter activity. This capability was lacking in case of transfection with ARE_del_IL22, containing a deleted fragment of the IL-22-3′-UTR without ARE (Fig. 5b). Interestingly, concomitant activation of Jurkat T cells by 12-O-tetradecanoylphorbol-13-acetate (TPA)/A23187 (calcium ionophore) nullified the inhibitory action of the IL-22-3′-UTR (Fig. 5c). In accord with a previous report^34^, constitutive expression of TTP was detectable by immunoblot analysis in Jurkat T cells. Levels of TTP in these cells were not further increased under the influence of TPA/A23187 (Fig. 5d). Data thus suggest that endogenously expressed TTP present in Jurkat T cells contributes to post-transcriptional gene regulation achieved by the IL-22-3′-UTR. SV40-driven target gene overexpression in HEK293 cells was employed to further address the role of TTP in gene regulation *via* the IL-22-3′-UTR. As shown in Fig. 5e, overexpression of human TTP reduced luciferase activity (when achieved through a plasmid containing ARE derived from the IL-22-3′-UTR – transfection with wt_UTR_IL22 or ARE37_IL22). In contrast, overexpression of TTP (see Fig. 5f) in combination with ARE_del_IL22 or with a luciferase expression plasmid entirely lacking IL-22-3′-UTR (and thus ARE) sequences did not inhibit but tended to increase luciferase reporter activity. This observation excludes suppressive effects of TTP overexpression acting on the level of luciferase enzyme transcription or activity.

**Figure 6:**
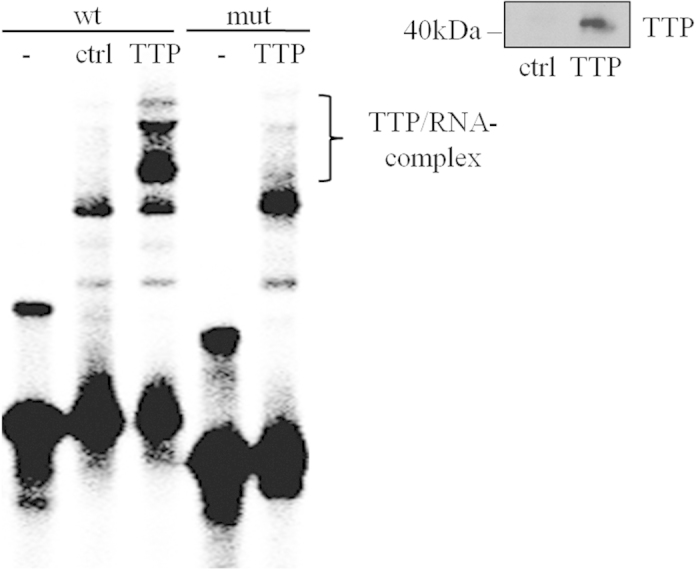
Binding of TTP to ARE located in the IL-22-3′-UTR as detected *in vitro* by RNA-EMSA. *In vitro* translated TTP was incubated together with a ^32^P-γ-ATP-labelled RNA oligonucleotide probe that includes the ARE5/6 region of the human IL-22-3′-UTR (see Fig. 1). In addition to this ‘wildtype’ (wt) oligonucleotide, a ‘mutated’ (mut) oligonucleotide was used lacking regular ARE sequences (see methods section). Reaction mixtures were subjected to native polyacrylamide gel electrophoresis. The brace indicates positions of retarded complexes present when the ‘wildtype’ but absent when the ‘mutated’ oligonucleotide was used. This retarded signal allegedly represents RNA/TTP complexes. Ctrl, denotes a control-*in vitro* translation setup with expression of an unrelated protein (firefly-luciferase) serving as control for unspecific protein/RNA interactions. Inset: Immunoblot analysis of *in vitro* translated TTP. One representative of three independently performed experiments is shown.

Finally, *in vitro* binding assays were performed that demonstrate physical binding of TTP to an RNA sequence derived from the IL-22-3′-UTR but not to a mutated counterpart (Fig. 6). This RNA oligonucleotide was specifically selected and spans the region of human ARE5/6. Notably, the whole IL-22-3′-UTR sequence covered by this RNA oligonucleotide (45 nt) is conserved between mice and humans displaying 93.3% identity. Data altogether indicate that TTP is able to regulate reporter gene expression by interacting with adjacent IL-22-3′-UTR sequences and thus by destabilizing target mRNA.

### The role of the MEK/ERK pathway in post-transcriptional regulation of IL-22 expression

**Figure 7:**
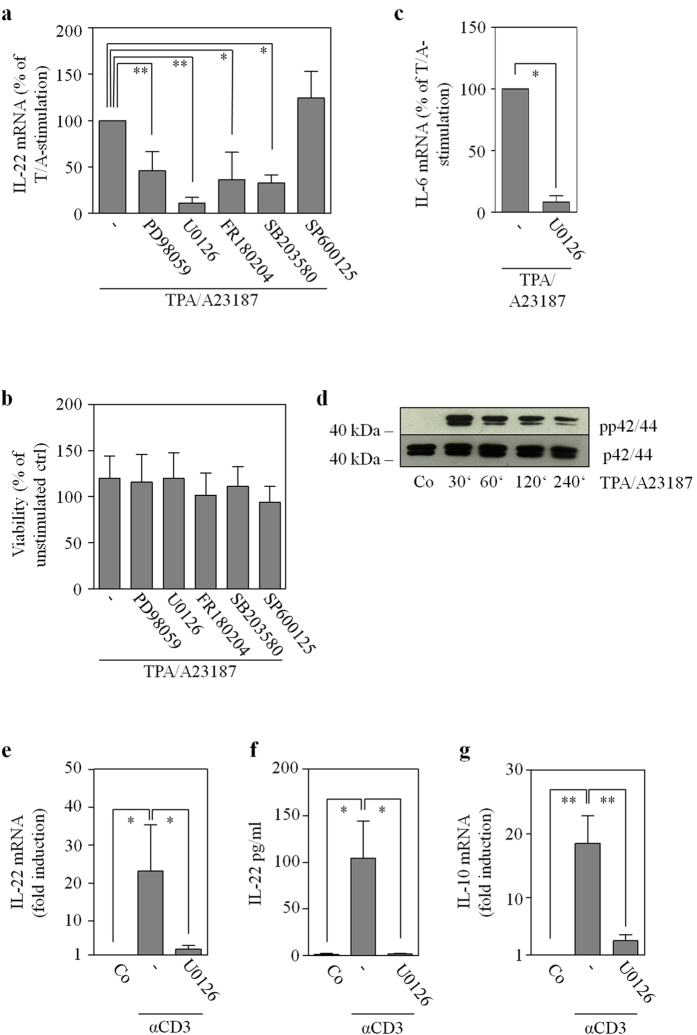
MAP kinase signaling is essential to IL-22 mRNA induction. (**a**–**c**) Where indicated, Jurkat T cells were pretreated with MAP kinase inhibitors for 1 h prior to stimulation with TPA (100 ng/ml)/A23187 (10 μM) for 4 h. PD98059 (n = 7), 50 μM; U0126 (n = 3), 10 μM, FR180204 (n = 11), 10 μM; SP600125 (n = 3), 10 μM; SB203580 (n = 5), 10 μM. In addition, cell were kept as unstimulated control. All cultures were adjusted to a final concentration of 0.21% DMSO (vehicle for TPA/A23187 plus inhibitor). (**a**,**c**) IL-22 and IL-6 mRNA were determined by realtime PCR. Target mRNA was normalized to GAPDH. Data are depicted as % of TPA/A23187-stimulation (means ± SD; *p < 0.05, **p < 0.01). Fold-induction of mRNA by TPA/A23187 (compared to unstimulated control): 121.12 for IL-22 (n = 16; p < 0.001), 19.3 for IL-6 (n = 3; p < 0.001). Statistical analysis on raw data, one-way analysis of variance with *post-hoc* Bonferroni-correction. (**b**) Viability is shown as % of untreated control (means ± SD). (**d**) Jurkat T cells were kept as unstimulated control or stimulated with TPA (100 ng/ml)/A23187 (10 μM). All cultures were adjusted to a final concentration of 0.11% DMSO (vehicle for TPA/A23187). After indicated time points, ERK (p42/44) activation, as detected by p42/44 phosphorylation, was determined by immunoblot analysis. One representative of three independently performed experiments is shown. (**e**–**g**) Primary human T cells were kept as unstimulated control (Co) or stimulated with αCD3 (20 μg/ml) for 24 h. Where indicated, cells were pretreated with U0126 (10 μM) for 1 h. All cultures were adjusted to a final concentration of 0.1% DMSO (vehicle for U0126). (**e**,**g**) IL-22 (e, n = 6) or IL-10 (g, n = 5) mRNA, determined by realtime PCR, was normalized to that of GAPDH (means ± SEM *versus* unstimulated control; *p < 0.05, **p < 0.01). Statistical analysis on raw data, one-way analysis of variance with *post-hoc* Bonferroni-correction. (**f**) IL-22 secretion was determined by ELISA. Data are shown as means ± SEM (n = 4; *p < 0.05). Statistical analysis, one-way analysis of variance with *post-hoc* Bonferroni-correction.

**Figure 8:**
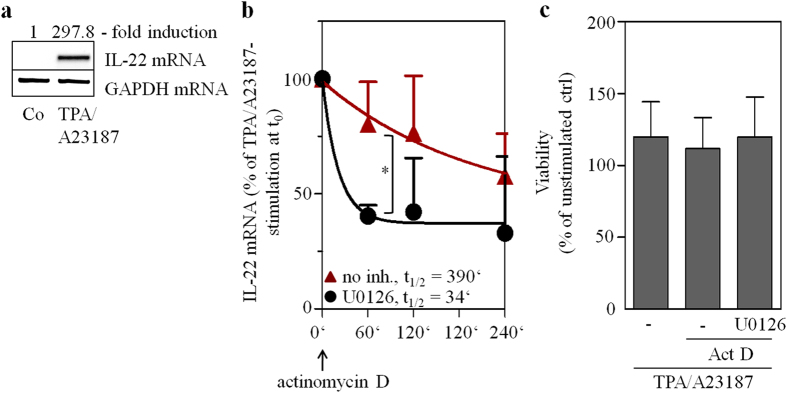
IL-22 mRNA decay is accelerated by U0126. (**a**–**c**) Jurkat T cells were either kept as unstimulated control (Co) or stimulated with TPA (100 ng/ml)/A23187 (10 μM) for 4 h. (**a**) Thereafter, IL-22 mRNA expression was assessed. One representative of three independently performed experiments is shown in which IL-22 mRNA is determined by standard PCR and realtime PCR (normalized to GAPDH with fold-induction *versus* control), respectively. All cultures were adjusted to a final concentration of 0.11% DMSO (vehicle for TPA/A23187). (**b**,**c**) After the 4 h induction period, cells were washed twice with PBS. Then actinomycin (Act) D (0.5 μg/ml) and, where indicated, U0126 (10 μM) was added. All cultures were adjusted to a final concentration of 0.22% DMSO (vehicle for TPA/A23187, Act D, U0126). (**b**) IL-22 mRNA levels were determined by realtime PCR at the indicated time points. IL-22 mRNA was normalized to that of GAPDH. Data depicted (as % of IL-22 mRNA expression after TPA/A23187 at t_0_, the time point of Act D addition) are expressed as means ± SD (n = 3; *p = 0.0215). Statistical analysis on percent data, Student’s t-test. (**c**) After a total incubation time of 8 h (4 h induction period using TPA/A23187 followed by 4 h of incubation with Act D in presence or absence of U0126), cell viability was determined and is shown as percent of untreated control (n = 3).

Recently, we reported on robust IL-22 mRNA and promoter induction detected in Jurkat T cells stimulated by TPA/A23187^22^ (see also Fig. 8a). Those studies^22^ likewise revealed suppression of TPA/A23187-induced IL-22 mRNA expression by the mitogen-activated protein kinase kinase (MEK)-1/2 inhibitor U0126^35^. In order to extend those previous data, TPA/A23187-activated Jurkat T cells were coincubated with a panel of pharmacological inhibitors affecting the mitogen-activated protein kinase (MAPK)/extracellular-signal-regulated kinases (ERK) signaling pathway. As shown in Fig. 7a, PD98059 (targeting MEK1/2 albeit with less potency compared to U0126) and FR180204 (targeting ERK1/2) as well as SB203580 (targeting p38 MAPK) but not SP600125 (targeting c-jun N-terminal kinases) significantly inhibited IL-22 mRNA expression. Those experiments were performed under conditions not affecting cell viability (Fig. 7b). Alike IL-22, also expression of related IL-6 was potently inhibited by U0126 in TPA/A23187-stimulated Jurkat T cells (Fig. 7c). Since U0126 was, by far, the most effective inhibitor of IL-22 expression (Fig. 7a and ref. 22) and TPA/A23187 potently activated the MEK/ERK pathway in Jurkat T cells (Fig. 7d), we chose to focus on this inhibitor in subsequent experiments. Notably, U0126 likewise suppressed IL-22 mRNA induction (Fig. 7e) and protein release (Fig. 7f) as well as IL-10 mRNA induction (Fig. 7g) by human αCD3-stimulated primary T cells.

Although MAPK signaling is mandatory for maximal cellular activation by NF-AT^36^ and thus likely involved in IL-22 promoter activation^22^, effects of U0126 on IL-22 mRNA stability were assessed in Jurkat T cells after a 4 h induction period using TPA/A23187. Figure 8a confirms robust IL-22 mRNA induction by TPA/A23187-stimulated Jurkat T cells^22^. As determined by actinomycin D-mediated transcriptional blockage performed in those same experiments, Jurkat T cells displayed significantly diminished IL-22 mRNA stability under the influence of U0126 (Fig. 8b). Those experiments were performed under conditions not affecting cell viability (Fig. 8c).

**Figure 9:**
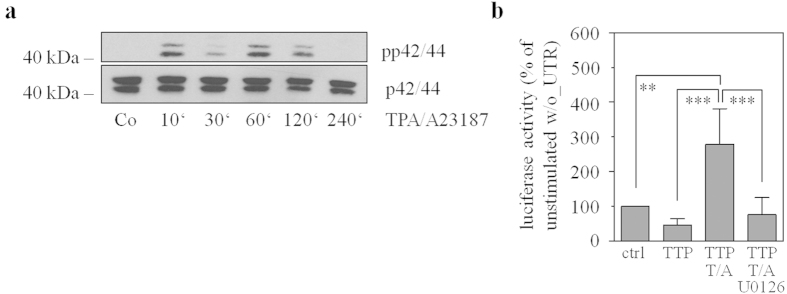
U0126 abolishes TPA/A23187-induced reporter gene upregulation in TTP overexpressing HEK293 cells. (**a**) HEK293 cells were either kept as unstimulated control or stimulated with TPA (100 ng/ml)/A23187 (10 μM) for indicated time periods. All cultures were adjusted to a final concentration of 0.11% DMSO (vehicle for TPA/A23187). Thereafter, ERK (p42/44) activation, as detected by p42/44 phosphorylation, was determined by immunoblot analysis. One representative of three independently performed experiments is shown. (**b**) HEK293 cells were transfected for 16 h with wt_UTR_IL22 luciferase reporter plasmid together with either a control (ctrl)- or a TTP-expression-plasmid. Thereafter, cells were stimulated as indicated with TPA (T, 100 ng/ml)/A23187 (A, 10 μM) in presence or absence of U0126 (10 μM) for 4 h. All cultures were adjusted to a final concentration of 0.21% DMSO (vehicle for T/A, U0126). Luciferase activity depicted (as % of unstimulated wt_UTR_IL22-/ctrl-plasmid transfection) is expressed as means ± SD (n = 4; **p < 0.01, ***p < 0.001). Statistical analysis on raw data, one-way analysis of variance with *post-hoc* Bonferroni correction.

Since TTP is a known target of the MEK/ERK pathway^25^, TPA/A23187 activates ERK1/2 in Jurkat T cells (Fig. 7d) as well as HEK293 cells (Fig. 9a), and the MEK1/2 inhibitor U0126 reduced IL-22 mRNA half-life in TPA/A23187-stimulated Jurkat T cells (Fig. 8b), effects of U0126 were investigated in the context of luciferase-reporter activity under the control of the IL-22-3′-UTR. For that purpose, HEK293 cells were transfected with wt_UTR_IL22 alone or in combination with the TTP expression plasmid. Cells were additionally stimulated with TPA/A23187 alone or in combination with U0126. As already shown (Fig. 5e), TTP overexpression reduced IL-22-3′-UTR-directed luciferase-reporter activity (Fig. 9b). Suppression by TTP was, in accord with data on Jurkat T cells (Fig. 5c), reversed under the influence of TPA/A23187 (Fig. 9b). Since TPA/A23187 did not significantly affect luciferase reporter activity in the absence of IL-22-3′-UTR (and thus ARE) sequences (111.7 ± 12.1% for TPA/A23187 *versus* unstimulated HEK293 cells with luciferase activity of unstimulated cells set as 100%, n = 3), stimulatory effects of TPA/A23187 acting on the level of luciferase enzyme transcription or activity can be excluded in these experiments. Notably, this TPA/A23187 effect was nullified by coincubation with U0126 (Fig. 9b). Data altogether suggest that the inhibitory action of TTP on IL-22-3′-UTR directed luciferase gene expression is counteracted by TPA/A23187-stimulated MEK/ERK signaling.

## Discussion

IL-6, IL-10, and IL-22 are related surrogates of immunoactivation with partly overlapping regulatory functions^37^. These cytokines display tissue protective properties^15^,^38^,^39^ that, however, come along with the potential to drive tumor growth^20^,^40^,^41^. This connects to immunosuppressive^37^,^41^ and oncogenic functions^42^ of STAT3 in leukocytes and cancerous cells, respectively. In addition, whereas functional IL-10 receptors lack on hepatocytes^43^, IL-6 and IL-22 are pivotal mediators of the STAT3-driven acute phase response^44^. Here, we characterize regulation by TTP as further IL-22 characteristic being shared with IL-6^25^,^29^ and IL-10^25^,^28^.

To specify the role of TTP for IL-22 expression, TTP^−/−^ mice that suffer from life-shortening harsh inflammation^31^ were investigated. We detected increased serum IL-22 in TTP^−/−^ mice as compared to wt littermates which was paralleled by a bias observed in TTP-deficient cultured splenocytes and isolated splenic CD3^+^ T cells to express elevated levels of IL-22. Data demonstrate the capability of TTP to modulate IL-22 expression in primary murine T cells. Notably, this conclusion is at variance with a previous report not observing upregulation of IL-22 secretion in TTP-deficient isolated T cells^32^. However, fundamental different protocols were used. The latter study used naïve CD4^+^ T cells differentiated in presence of αCD3/αCD28 towards T0, Th1, Th17, and Th22 using 3-day-incubation-protocols. Thereafter, IL-22 secretion was detectable only in Th22 cells and did not differ between genotypes^32^. Herein, we specifically decided to use whole splenic CD3^+^ T cells that include memory T cell subsets prone to efficiently express IL-22. After only 4 h of stimulation by αCD3/αCD28, brisk upregulation of IL-22 mRNA was detected. This brief incubation period precludes indirect effects of extended stimulation protocols and enables efficient determination of IL-22 mRNA half-life. In fact, splenic TTP^−/−^ CD3^+^ T cells displayed significantly prolonged IL-22 mRNA half-life in the context of αCD3/αCD28 stimulation.

Using Jurkat T and HEK293 cells, luciferase reporter assays were performed in order to deepen knowledge on the relevance of the IL-22-3′-UTR for IL-22 expression. Experiments revealed a strong ARE-dependent mRNA destabilizing potential of the IL-22-3′-UTR that was nullified in response to TPA/A23187. Notably, overexpression of TTP inhibited luciferase activity under the control of the IL-22-3′-UTR. *In vitro* assays furthermore demonstrated physical binding of TTP to conserved ARE within the human IL-22-3′-UTR. Altogether, data indicate that TTP directly regulates IL-22 in activated T cells. Notably, functional TTP is constitutively expressed in Jurkat T cells^34^ and, after polyclonal activation, rapidly induced and biological active in primary human T cells^45^,^46^.

Previously, we observed in TPA/A23187-stimulated Jurkat T cells suppression of IL-22 mRNA by the MEK1/2 inhibitor U0126^22^. To expand this observation, a panel of MAP kinase inhibitors was evaluated herein. With the exception of SP600125, all compounds reduced IL-22 mRNA. Among those inhibitors, which included the p38 MAP kinase inhibitor SB203580, U0126 was the most potent. The specificity of this U0126 action is emphasized by the notion that PD98059, also targeting MEK1/2, and FR180204, targeting the MEK1/2 downstream kinases ERK1/2^47^, were as well capable of modulating IL-22 mRNA in Jurkat T cells. U0126 likewise suppressed production of IL-22 by primary human CD3^+^ T cells, along with that of related IL-10. Observations correspond to a recent report demonstrating inhibition of IL-22 secretion by PD98059 as detected in Th17 cells generated from naïve T cells under the influence of IL-1β, IL-6, and IL-23 as well as anti-IFNγ and anti-IL-4. However, the capacity of MEK1/2 inhibition to directly impair IL-22 gene expression was not assessed^48^.

Activation of the MEK/ERK axis, particularly in cooperation with p38 MAP kinase, has the capability to target and inhibit TTP function^25^,^49^,^50^,^51^. Modulation of TTP biological function by phosphorylation is supposed to be counteracted by the phosphatase PP2A^25^. Actually, we observed significant inhibition of IL-22 mRNA half-life by U0126 as detected in TPA/A23187-stimulated Jurkat T cells. Notably, ERK1/2 (Fig. 7d) as well as p38 MAP kinase^52^ are being activated in Jurkat T cells under those experimental conditions. A regulatory interplay between the MEK/ERK axis and TTP was substantiated by performing luciferase reporter assays in TTP-overexpressing HEK293 cells. Under those conditions, U0126 reversed transcript stabilization achieved by TPA/A23187. Data demonstrate that the MEK/ERK pathway has the capability to antagonize TTP functions that aim at destabilizing IL-22 mRNA. In light of a broader context, promoting IL-22 expression feeds into the role of the MEK/ERK axis to support induction of genes associated with immunoactivation and inflammation^47^.

Altogether, this is the first report indicating that TTP by interaction with the IL-22-3′-UTR directly regulates IL-22 gene expression in a T cell autonomous fashion. Although not addressed herein, it tempting to speculate that IL-22 regulation by TTP is part of an interdependent regulatory network enforcing posttranscriptional regulation by diverse mechanisms comprising of further ARE-binding proteins such as human antigen R (HuR), KH-type splicing regulatory protein (KSRP), and AU-binding factor 1 (AUF1) as well as microRNA populations^23^,^24^. This layer of gene regulation may open the avenue towards novel pharmacological approaches. In that context it is noteworthy that pharmacological regulation of TTP availability by using an oligonucleotide targeting insulin receptor substrate-1 associates with modulation of endothelial cell steady-state levels of a set of ARE-containing mRNA molecules coding for TNFα, vascular endothelial growth factor, (VEGF), IL-1β, IL-8, IL-12, and IL-22^53^. However, the biochemical basis of this association, specifically the relationship between TTP and IL-22 mRNA stability as well as interactions between TTP and the IL-22-3′-UTR, was not addressed in that report.

Posttranscriptional regulation as detected herein is frequently organized in functional units^30^. In case of TTP, control of pro-inflammatory genes prevails which is clearly documented *in vivo* by overwhelming TNFα- and IL-23-dependent inflammation in TTP^−/−^ mice^31^,^32^. Beyond that, it has become evident that TTP is able to inhibit in quasi coordinated manner a set of cytokines and growth factors that are supposed to promote diverse aspects of carcinogenesis^25^,^54^. Those include obvious candidates such as vascular endothelial growth factor^55^, IL-1^56^, IL-8^57^, IL-6^40^, IL-10^41^, and IL-22^20^. Interestingly, expression of TTP is characteristically low in cancerous tissue^54^. Data presented herein indicate that therapeutic strategies aiming at upregulation of TTP biological activity may, among others, oppose the ill-fated role of IL-22 in colon, liver, lung, and skin carcinogenesis^20^,^58^,^59^,^60^.

## Methods

### Reagents

Murine IL-12 and IL-18 were from Peprotech Inc. (Frankfurt, Germany), anti-murine-CD3, anti-murine-CD28, and anti-human-CD3 antibodies were from BioLegend (San Diego, CA, USA). Inhibitors U0126, PD98059, SB203580, FR180204, and SP600125 were from Calbiochem (Schwalbach, Germany). A23187 was from AppliChem (Karlsruhe, Germany), TPA was from Enzo Life Sciences, (Lörrach, Germany), and actinomycin D was from Sigma-Aldrich (Taufkirchen, Germany).

### Animals

All mice were housed in accordance with standard animal care requirements and maintained under specified pathogen-free conditions on a 12/12-h light/dark circle. Throughout the study 14–16 week-old mice were used. Water and food were given *ad libitum*. TTP^+/−^ mice (a kind gift by Dr. Blackshear^27^, NIEHS, National Institutes of Health, Research Triangle Park, NC, USA) had a C57BL/6 background. TTP^−/−^ and TTP^+/+^ mice were obtained by mating TTP^+/−^ animals. Genotyping of mice was performed by polymerase chain reaction (PCR), using primers that span the regions of the wild type genes disrupted by the targeting vectors. The following oligonucleotides (Sigma-Aldrich) were used for genotyping the *Ttp* locus: TTP-wt/ko-for, 5′-GAGGGCCGAAGCTG CGGTGGGT-3′; TTP-wt-rev, 5′-GGCTGGCCAGGGAGAGCTAGGTC-3′; and TTP-ko-rev, 5′-CTGTTGTGCCCAGTCAT AGCCG-3′. Animal studies were performed in accordance with German animal protection law.

### Cultivation of HEK-293 cells, Jurkat T cells, human primary T cells, murine splenocytes, and murine splenic CD3^+^ T cells

Cell culture was performed at 37 °C and 5% CO_2_. *Human Jurkat T cells* – Jurkat T cells were obtained from the American Type Culture Collection (Manassas, VA) and cultured in RPMI 1640 (Life Technologies, Darmstadt, Germany) supplemented with 100 units/ml penicillin, 100 μg/ml streptomycin, and 10% heat-inactivated FCS (Life Technologies). For experiments, Jurkat T cells were seeded on 6-well polystyrene plates (Greiner, Frickenhausen, Germany) at a density of 2.5 × 10^6^ cells/ml. To assess viability of Jurkat T cells WST assays were performed in 96-well polystyrene plates (Greiner, 3000 cells in 100 μl medium) in parallel to stimulation experiments. After treatment, 10 μl of WST reagent (Roche, Mannheim, Germany) were added and viability was analyzed according to the manufacturer’s instructions. WST assays were verified for linearity. *HEK293 cells –* HEK293 embryonic kidney cells (German Collection of Microorganisms and Cell Cultures; Braunschweig, Germany) were maintained in DMEM supplemented with 100 units/ml penicillin, 100 μg/ml streptomycin, and 10% heat-inactivated FCS (Life Technologies). For experiments, HEK293 cells were seeded on 6-well polystyrene plates (Greiner) in the aforementioned culture medium. *Human CD3*^*+*^ *T cells* – For isolation of peripheral blood mononuclear cells (PBMC), written informed consent was obtained from healthy donors, and blood was taken. All experimental protocols were approved by the ‘Ethik Kommission’ of the University Hospital Goethe-University Frankfurt. The methods were carried out in accordance with the approved guidelines. Healthy donors had abstained from taking drugs/medication for 2 weeks before the study. PBMC were isolated from peripheral blood using Histopaque-1077 (Sigma-Aldrich) according to the manufacturer’s instructions. The untouched CD3^+^ T cell population of PBMC was isolated using the Pan-T-cell isolation kit according to the manufacturer’s instructions (Miltenyi, Bergisch Gladbach, Germany). Cells were resuspended in RPMI 1640 supplemented with 10 mM HEPES, 100 units/ml penicillin, 100 μg/ml streptomycin, and 1% human serum (Life Technologies) and seeded at 3 × 10^6^ cells/ml in round-bottom polypropylene tubes. To assess successful isolation, FACS analysis (FACS Canto, BD Biosciences, Heidelberg, Germany) was performed with the following antibody: mouse monoclonal anti-human CD3-PerCP/Cy5.5 (BioLegend). CD3^+^ T cell isolation resulted in a mean purity of 98.2 ± 1.7% (n = 6). *Murine splenocytes and splenic CD3*^*+*^ *T cells* – Isolation of splenocytes from TTP^−/−^ mice and wt littermates as well as further isolation of untouched CD3^+^ T cells (Pan-T-cell isolation kit, Miltenyi) was performed according to the manufacturer’s instructions. For experiments, 5 ×10^6^ CD3^+^ T cells or splenocytes were seeded on 12-well polystyrene plates in RPMI 1640 culture medium supplemented with 10% FCS, 100 units/ml penicillin, and 100 μg/ml streptomycin. CD3^+^ cell isolation was evaluated by FACS analysis (FACS Canto) using hamster monoclonal anti-mouse CD3-PerCP/Cy5.5 (BioLegend). Purity of CD3^+^ cells was 97.9 ± 1.5% (n = 30).

### Cloning of the human IL-22-3′-UTR, transient transfection of Jurkat T cells and HEK293 cells, and luciferase reporter assays

To generate luciferase reporter constructs, we amplified 3′ flanking regions of the IL-22 mRNA (NM_020525) from cDNA generated from Jurkat T cell mRNA, using Phusion polymerase (Thermo Scientific, Waltham, USA). The following primers (excluding an additional NotI cloning site) were used: wt_UTR_IL22 (553 bp), ARE_del_IL22 (206 bp): forward 5′-CCAGAGCAAAGCTGAAAAATG-3′; ARE37_IL22 (206 bp): forward 5′-GTTTCCATAATCAGTACTTTATATTTATAA-3′. The reverse primers (excluding an additional flanking XhoI cloning/restriction site) were: wt_UTR_IL22, ARE37_IL22: reverse 5′-GGATATCCAAGTGTTTATTGAGG-3′; ARE_del_IL22: reverse 5′-TATGCTTAGAAAGTCTACC-3′. Fragments were cloned into psiCheck2 (Promega, Mannheim, Germany) and sequenced thereafter (MWG, Ebersberg, Germany). *Transfection of Jurkat T cells* – psiCheck2-plasmids were transiently transfected into Jurkat T cells using DMRIE-C reagent (Life Technologies). For each reaction, 4 μg of indicated plasmid were transfected into 2.5 × 10^6^ Jurkat T cells according to the manufacturer’s instructions. The transfection was stopped after 5 h by adding 2 ml of Jurkat T culture medium (aforementioned) supplemented with 5% heat-inactivated FCS. After 16 h of rest, cells were harvested, further kept as unstimulated control or stimulated as described in the respective figure legend and harvested thereafter. *Transfection of HEK293 cells* – 24 h before transfection, HEK293 cells were seeded on Poly-L-lysine (Sigma-Aldrich) coated 6-well polystyrene plates (Greiner) in the aforementioned culture medium. Transfection was conducted with 0.5 μg of luciferase reporter plasmid and 2 μg of SV40-driven pZeo_hTTP expression plasmid^61^ or empty vector as indicated using Lipofectamine2000 (Life Technologies) according to the manufacturer’s instructions. *Determination of luciferase activity* – After 16 h of resting, cells were harvested or further kept as unstimulated control or stimulated as described in the respective figure legend and harvested thereafter. Luciferase activity was determined using the dual luciferase reporter gene system (Promega) and an automated chemiluminescence detector (GloMax®, Promega).

### Analysis of IL-22 and glyceraldehyde-3-phosphate-dehydrogenase (GAPDH) mRNA by standard PCR

Total RNA, isolated by Tri-Reagent (Sigma-Aldrich) was transcribed using random hexameric primers (Qiagen, Hilden, Germany) and Moloney virus reverse transcriptase (Life Techno-logies). The following sequence was performed for each PCR reaction: 95 °C (10 min, 1 cycle); 95 °C (30 sec), 60 °C (GAPDH) or 58 °C (IL-22) for 30 sec, and 72 °C for 1 min (with 25 cycles for GAPDH and 38 cycles for IL-22); and a final extension phase at 72 °C (7 min). Primers: IL-22, forward: 5′-CACGGAGTCAGTATG AGTGAG-3′, reverse: 5′-CAAATGCAGGCATTTCTCAGAGA-3′; GAPDH, forward: 5′-ACCACAGTCCATGCCATCAC-3′, reverse: 5′-TCCACCACCCTGTTGCTGTA-3′. Amplicon length: IL-22, 299 bp; GAPDH, 452 bp.

### Detection of IL-22, IL-6 and IL-10 mRNA by realtime PCR

Total RNA, isolated by Tri-Reagent (Sigma-Aldrich) was transcribed using random hexameric primers (Qiagen) and Moloney virus reverse transcriptase (Life Technologies). During realtime PCR, changes in fluorescence are caused by the Taq polymerase degrading a probe containing a fluorescent dye (GAPDH, hs-IL-22: VIC; all others: FAM). Pre-developed reagents: hs-GAPDH (4310884E), hs-IL-22 (Hs01574152_g1), hs-IL-6 (Hs00985639_m1), hs-IL-10 (Hs99999035_m1), mm-GAPDH (4352339E), mm-IL-22 (Mm00444241_m1) (Life Technologies). Assay mix was from Life Technologies. Realtime PCR (AbiPrism7500 Fast Sequence Detector, Life Technologies): Two initial steps at 50 °C (2 min) and 95 °C (20 sec) were followed by 40 cycles at 95 °C (3 sec) and 60 °C (30 sec). Detection of the dequenched probe, calculation of threshold cycles (C_T_ values), and data analysis were performed by the Sequence Detector software. Relative changes in mRNA expression compared to unstimulated control and normalized to GAPDH were quantified by the 2^−ΔΔCT^ method.

### Immunoblot analysis

Whole cell lysates were generated using lysis buffer (150 mM NaCl, 1 mM CaCl_2_, 25 mM Tris-Cl (pH 7.4), 1% Triton X-100), supplemented with protease inhibitor cocktail (Roche Diagnostics) and DTT, Na_3_VO_4_, PMSF (each 1 mM), and NaF (20 mM). Thereafter, SDS-PAGE and immunoblotting were performed. For detection of total ERK, blots were stripped and reprobed. To detect TTP and β-tubulin on the same blot, the blot was cut. Antibodies (against human antigen): phospho (Y202/Y204)-ERK, rabbit polyclonal antibody (p-p42/44); total ERK, rabbit polyclonal antibody (Cell Signaling, Frankfurt, Germany); TTP, rabbit monoclonal antibody (Abcam plc, Cambridge, UK); β -tubulin, mouse monoclonal antibody (Santa Cruz Biotechnology Inc., Heidelberg, Germany).

### Cytokine release detected by enzyme-linked immunosorbent assay (ELISA)

Human and murine IL-22, murine IL-6 (DuoSet ELISA, R&D-Systems, Wiesbaden, Germany), and murine IL-10 (eBioscience, Frankfurt am Main, Germany) in culture supernatants were determined by ELISA. Quantikine ELISA (R&D-Systems) was used to detect serum murine IL-6, IL-10, and IL-22. Assays were performed according to the manufacturers’ instructions.

### RNA-electrophoretic mobility shift assay (EMSA)

Human TTP or control protein (firefly luciferase) were *in vitro* translated using the TNT-Quick-coupled-*in vitro* transcription/translation system (Promega). Single stranded RNA-oligonucleotides (Biomers, Ulm, Germany) were radiolabelled using T4 polynucleotide kinase (Roche) and ^32^P-γ-ATP (Perkin Elmer, Baesweiler, Germany). The following RNA-oligonucleotides were used: 5′-GCAUUUUAUUUAUAUCAUUUUAUUAAUAUGGAUUUAUUUAUAGAA-3′ (wt), 5′-GCAUUUUAGCAUAUCAUUUUAUUAAUAUGGAGCAGCAUAGAA-3′ (mut). For binding reactions, RNA-oligonucleotides (75000 cpm/reaction) were incubated with *in vitro* translated protein in REMSA-buffer (10 mM HEPES, 20 mM KCl, 1 mM MgCl_2_, 1 mM DTT, 200 ng/ml yeast tRNA) for 20 min at room temperature. The complexes were separated on a native 4.5% polyacrylamide gel and run in Tris-borate-EDTA buffer. Gels were fixed, vacuum dried and signals were visualized using a phosphorimager and detection software.

### Statistical analysis

Data are shown as means ± standard deviation (SD, cell culture data using cell lines) or as means ± standard error of the mean (SEM, animal data or cell culture data using primary cells) and presented as pg/ml, fold-induction, or percent (viability, IL-22 mRNA or luciferase activity relative to the indicated control). Statistical analysis was performed as indicated in the legends by one-way analysis of variance with *post-hoc* Bonferroni correction (for multi-comparisons) or unpaired Student’s t-test. Differences were considered statistically significant if the p value was <0.05 (Prism 5.0, GraphPad, La Jolla, CA, USA). For analysis by unpaired Student’s t-test specific p values are additionally indicated in the legends whereas in case of analysis by one-way analysis of variance upper only limits of p values are depicted. IL-22 mRNA half-life was calculated using nonlinear regression analysis (Prism, GraphPad).

## Additional Information

**How to cite this article**: Härdle, L. *et al.* Tristetraprolin regulation of interleukin-22 production. *Sci. Rep.* **5**, 15112; doi: 10.1038/srep15112 (2015).
