# Erratum: Tristetraprolin regulation of interleukin-22 production

**DOI:** 10.1038/srep17160

**Published:** 2015-12-18

**Authors:** Lorena Härdle, Malte Bachmann, Franziska Bollmann, Andrea Pautz, Tobias Schmid, Wolfgang Eberhardt, Hartmut Kleinert, Josef Pfeilschifter, Heiko Mühl

Scientific Reports 5 Article number: 15112; 10.1038/srep15112 published online: 10212015; updated: 12182015.

The original HTML version of this Article incorrectly listed Wolfgang Eberhardt, Josef Pfeilschifter and Heiko Mühl as being affiliated with ‘Department of Pharmacology, University Medical Center of the Johannes-Gutenberg University Mainz, Mainz, Germany.’ The correct affiliation is listed below:

pharmazentrum frankfurt/ZAFES, University Hospital Goethe-University Frankfurt, Germany

In addition, Heiko Mühl was omitted as a corresponding author.

These errors have now been corrected in the HTML; the PDF version of the paper was correct from the time of publication.

